# Preliminary report of drive-through screening COVID-19 screening process in a large suburban community

**DOI:** 10.1186/s12245-021-00340-1

**Published:** 2021-03-24

**Authors:** Anne Ledvina, Ronny Otero, Jessica Hamilton, Carol Clark, Aveh Bastani, James Ziadeh, Jeffrey Ditkoff, Robert Swor

**Affiliations:** 1 Oakland University William Beaumont School of Medicine, Rochester, USA; 2 Department of Emergency Medicine, Beaumont Hospital-Royal Oak, Oakland University William Beaumont School of Medicine, 3601 W. 13 Mile Rd, Royal Oak, MI 48073 USA; 3 Department of Emergency Medicine, Beaumont-Troy Hospital, Troy, Mi USA

## Introduction

The 2019-novel coronavirus (2019-nCoV) was first identified in Wuhan China in early December, 2019, and named COVID-19 by the World Health Organization (WHO) on January 12, 2020. The first US COVID-19 case was identified in an urgent care clinic in Snohomish County, WA [1–3]. On March 10, 2020, the World Health Organization declared a global pandemic due to COVID-19 [4].

Patients with COVID-19 symptoms rapidly started to appear in US hospital emergency departments (ED), and these departments needed to quickly develop strategies to respond to this sudden influx of acutely ill and worried well patients. At the University of California-San Diego, two EDs tested for COVID-19 and reported their results during the first 10 days of screening in March, 2020. High-risk patients, as defined by the Centers for Disease Control (fever, cough, shortness of breath and travel to an endemic area, healthcare worker with exposure), were offered screening. Of the 283 tests ordered, 10% were COVID-19 positive [5].

As cases of COVID-19 grew exponentially, ED volumes in our region also rapidly increased, and concern was raised that hospitals would not be able to provide care for these patients. We sought out strategies to decompress the volume of low acuity patients in the ED by screening them outside of the hospital [6, 7]. Such screening took place in Africa during the Ebola crisis of 2014–2016. One hospital, Redemption Hospital (Liberia), created an out-of-hospital screening program to decrease patients entering the hospital and identify patients at risk for Ebola [8, 9].

Early in the COVID-19 pandemic, providers in Korea created drive-through (DT) 2019-nCoV testing centers. This was implemented in a remote, large parking lot. The cars followed a designated sequence to allow examination, specimen collection, and instruction. This DT testing method was introduced at Kyungpook National University Chilgok Hospital, Daegu, Korea, on February 23, 2020, and later expanded to at least an additional 68 testing centers [10].

There is currently a dearth of literature evaluating the safety and outcomes of out-of-hospital testing for COVID-19 as a means to decompress the healthcare system and provide large scale epidemiological screening. For this reason, the objective of this investigation was to describe testing and clinical outcomes of patients evaluated by a hospital system-based DT screening strategy early in the 2020 COVID-19 pandemic.

## Methods

### Settings and population

We performed a retrospective study of a DT screening process conducted at Beaumont Health System hospital system. The study was conducted at eight hospitals of the Beaumont Health System that provides care to an estimated population of 30% of Southeastern Michigan, a community of 4.7 million people. The EDs for the system had over 570,000 ED visits during 2019 [11].

### Screening procedure

#### General screening procedure

Screening occurred between March 12 and April 15, 2020. The curbside screening took place at a hospital entrance adjacent to the ED. Incoming ED patients were screened predominantly by an advanced practice provider prior to the patient leaving their vehicle. Standardized, EMTALA-compliant criteria were utilized for screening, and patients were triaged to ED evaluation or (with their consent) to curbside screening. Some patients were insistent upon utilizing drive through, rather than ED assessment.

After triage, patients were directed in their car to a separate hospital entrance for further evaluation as has been previously described [12]. They were evaluated with initial vital signs including pulse oximetry. After medical screening exam was performed, testing was completed or not based on current protocol and availability of testing. Patients were either referred to the ED or discharged home with education regarding quarantine and return precautions. As the scope of the COVID-19 pandemic evolved, screening questions and testing strategy changed to identify and test patients at high risk for COVID-19 infection (cough, fever, SOB). Asymptomatic patients were not tested. There was also a need to screen patients with a clinical syndrome consistent with COVID-19, to identify those with severe disease and the need for ED evaluation. Microbiological testing changed during the study period based on availability of testing and testing media. Testing strategy varied by the day, which was reflective of national shortages of testing capacity. An initial phase of aggressive COVID-19 testing was started, which was limited by test capacity, and subsequently was reserved for those who were symptomatic with comorbidities. In the final week of the study, only clinical evaluations were performed at DT without the option of COVID-19 testing. After evaluation, patients were referred for further ED care or discharged with COVID-19 specific instructions.

#### Data collection

We queried the health system electronic medical record Epic^TM^ (Madison, WI) database to confirm that patients received a curbside evaluation. Patient demographics including age, gender, and race were abstracted as were initial vital signs obtained, and, if performed, results of COVID-19 testing were recorded. Testing was processed initially by the State of Michigan Public Health laboratory and migrated to a local hospital lab during the study. Patients were subsequently notified by phone of positive test results.

### Outcomes

The primary outcome was (1) the proportion of patients who returned for ED evaluation subsequent to an initial drive-through evaluation, (2) whether they were admitted to the hospital on the return visit, and (3) the hospital outcome. To obtain data regarding mortality post-screening of DT patients, a search of state mortality and probate records was conducted to identify mortality within 30 days of the index visit using a third-party vendor [13]. Secondary outcomes included microbiological outcomes of curbside screening tests and patient outcomes upon ED visit (discharged from ED, admitted to hospital, hospital discharge, and expiration while in the hospital). We reported frequencies and proportions of screened patient outcomes. Proportions were tested using Chi-square analysis and continuous variables using *t* test. The study was approved by the health system institutional review board.

## Results

A total of 7061 patients from all hospitals were screened (range 12–2660 patients/hospital) of which 58.0% were female and 48.0% Caucasian. The average age of subjects was 44.1 years old (range 0–97). A small number (*n*=111) were < 18 years old. Abnormal vital signs were identified in 1633 (23.1%) patients on initial DT. Of those 296 (4.2%) had fever ≥ 100.4 °F, 1355 (19.2%) with tachycardia (HR>100 bpm), 174 (2.5%) with hypoxia (SpO_2_ <94%), 7 (0.00%) with hypotension (Sys BP< 90), and 26 (0.4%) had tachypnea (RR > 24). Summary data is presented in Table 1.

**Table 1:** Subject demographics

Study subjects	*N*	%
Female	4087	57.8%
Male	2974	42.1%
Age (mean, range)	44.1 (0–97)	
Age < 18 years	111	1.6%
COVID-19 test performed	1407	19.9%
COVID positive	381	27.1%
Race		
Caucasian	3395	48.1%
African American	3045	43.1%
Asian	87	1.2%
Other	531	4.8%
Missing	3	
Vital signs		
T max (>100.4)	296	3.6%
SpO_2_≤ 94	174	2.5%
HR>100	1355	19.6%
RR>24	26	0.3%

Testing was done for COVID-19 in 1407 (19.9%) patients during the initial DT screening of whom 381 (27.1%) were positive for COVID-19. The median (IQR) time from testing to results obtained was lengthy, 9 (6, 12) days. There was no difference in the proportion tested by gender (males, 19.9% vs females, 19.9%). Testing was more common in patients with increased age (45.9 vs 43.6 years, *p*=0.001). There was a significant difference in proportion testing by race (Caucasian 23.9% vs Asian 19.5% vs African American (14.4%, *p*< 0.001).

An in-system ED revisit occurred in 688 (9.4%) patients with a median (IQR) time from initial DT evaluation of 4.3 (2.1, 6.5) days. Returning patients were admitted 27.7% of the time (195/668). Patients that returned to the ED were older (47.5 vs 43.7 years, *p*<0.001), more often African American compared to Caucasian ((11.7% vs 7.8%), *p*<0.001). There was no difference in rate of return by gender. Revisit rates were slightly decreased when testing was done during the initial drive through (7.9% vs 9.9%, *p*=0.03). System medical record review and statewide screening of mortality records identified that 17 (0.2%) of patients ultimately died after screening (Table 2).

**Table 2:** Subjects who were initially screened in DT and subsequently expired

Sex	Race	Age	COVID-19 test done on initial visit	Index visit-revisit (in days)^a^	Index visit-death (in days)	Abnormal vitals on drive through
Female	Black	63	Detected	4.8	9	None
Female	White	61		4.1	14	Febrile
Female	Black	58		1.7	12	Tachycardia
Female	Black	65	Detected	2.6	6	Febrile, tachycardia
Male	Black	77	Detected	1.9	16	None
Male	Black	71	Detected	5.6	16	Missing RR
Male	Black	97		3.9	20	Missing RR
Female	Black	82		1.1	5	None
Male	White	73		0.4	16	None
Male	White	55	Detected	n/a	4	Fever, tachycardia
Male	Black	80	Detected	n/a	12	None
Male	Black	35	Detected	n/a	10	None
Female	Black	42		n/a	19	None
Male	Black	71		n/a	9	None
Female	Black	68		n/a	16	Fever
Female	White	85		n/a	7	Hypoxia
Male	White	57		n/a	20	None

^a^n/a reflects no return visit to system hospital but identified on mortality review

## Discussion

This study demonstrates that this DT COVID-19 screening process effectively triaged patients presenting to the health system. However, almost 10% of patients subsequently returned to the ED, and a small percentage of those patients expired. This is the first study which addresses clinical outcomes of what has now become a ubiquitous intervention around the world. While this DT process has been adopted in many communities, it has evolved as the prevalence of disease and available resources have changed.

The DT screening process implemented in this system served an important role in responding to the early stage of the pandemic. First, it diverted patients from EDs which were struggling to cope with acutely ill COVID-19 patients. As many as 2660 patients would have presented to a study hospital over a 4-week study period. We identified that screening in the absence of testing was also useful in that greater than 90% of patients screened without testing did not return for ED care. There was a large demand for COVID-19 testing in the community and a concomitant statewide shortage of testing capacity and a need to triage patients that might most benefit from testing. We also needed to augment ED triage processes which were overwhelmed by marked increases in demand. This process was implemented during a time period with a high prevalence of disease in our region with associated substantial morbidity. The population screened was at higher risk of disease than other implemented programs, which increased the difficulty of accurate clinical assessment and triage of these patients. This study documents the limitations of the DT process and identifies the need for data to more fully evaluate its effectiveness. Although we have discontinued this DT process, it may well need to be implemented again in our community and many others as the COVID-19 pandemic continues.

This study is imperfect but reflects the state of the art in the early stages of the COVID-19 pandemic in the USA and this community. The availability of testing varied over the time course of this study and continues to vary in its availability and ability to provide timely results. We observed differences in the rates of testing by age and race, which are worthy of further scrutiny in subsequent studies on this topic. Only a small proportion of screened patients were actually tested, despite a high prevalence of disease. The proportion of revisits, admissions, and deaths are reflective of the high positive test rate. DT programs implemented later in the pandemic with readily available testing should have a lower rate of disease, but our data suggests that surveillance on outcomes should be performed. Our observation regarding the proportion of ED return visits is limited by being able to only identify return visits within our health care system. We were however able to identify deaths that occurred within the State of Michigan within 30 days after the index visit. We did not specifically identify the ED diagnosis of return visits. However, ED volumes in the entire state plummeted during this phase of the pandemic, and most hospital admissions during this time period were COVID related. Our study only assessed clinical outcomes of subjects and did not address the classic public health value associated with testing, index case identification, contact tracing, and quarantine. These public health outcomes are important, but unfortunately, they were not available in our community during this phase of the pandemic, as testing results took days to obtain.

## Conclusion

DT screening with or without testing for COVID-19 was an effective tool to decompress hospitals and contribute to surge management during this phase of the pandemic. This study identified that almost 10% of screened patients became very ill and required repeated emergency evaluation with complications including hospital admission and death. Drive-through screening programs have become a ubiquitous part of the community response to COVID-19. These data identify that DT screening programs should include measures to evaluate the safety of these programs.

## Data Availability

The datasets used and/or analyzed during the current study are available from the corresponding author on reasonable request.
